# Linking solid-state phenomena via energy differences in ‘archetype crystal structures’

**DOI:** 10.1107/S2052252524002641

**Published:** 2024-04-16

**Authors:** B. Dittrich, L. E. Connor, F. P. A. Fabbiani, P. Piechon

**Affiliations:** a Novartis Campus, Novartis Pharma AG, Postfach, Basel CH-4002, Switzerland; b Mathematisch Naturwiss. Fakultät, Universität Zürich, Winterthurerstrasse 190, Zürich CH-8057, Switzerland; ESRF, France

**Keywords:** quantum crystallography, archetype crystal structures, structure-specific restraints, quantum chemical energy differences, twinning

## Abstract

Categorization underlies understanding. Conceptualizing solid-state structures of organic molecules with ‘archetype crystal structures’ bridges established categories of disorder, polymorphism and solid solutions and is herein extended to special position and high-*Z*′ structures. The concept was developed in the context of disorder modelling [Dittrich, B. (2021). *IUCrJ*, **8**, 305–318] and relies on adding quantum chemical energy differences between disorder components to other criteria as an explanation as to why disorder – and disappearing disorder – occurs in an average structure. Part of the concept is that disorder, as probed by diffraction, affects entire molecules, rather than just the parts of a molecule with differing conformations, and the finding that an *R·T* energy difference between disorder archetypes is usually not exceeded. An illustrative example combining disorder and special positions is the crystal structure of oestradiol hemihydrate analysed here, where its space-group/subgroup relationship is required to explain its disorder of hydrogen-bonded hydrogen atoms. In addition, we show how high-*Z*′ structures can also be analysed energetically and understood via archetypes: high-*Z*′ structures occur when an energy gain from combining different rather than overall alike conformations in a crystal significantly exceeds *R·T*, and this finding is discussed in the context of earlier explanations in the literature. Twinning is not related to archetype structures since it involves macroscopic domains of the same crystal structure. Archetype crystal structures are distinguished from crystal structure prediction trial structures in that an experimental reference structure is required for them. Categorization into archetype structures also has practical relevance, leading to a new practice of disorder modelling in experimental least-squares refinement alluded to in the above-mentioned publication.

## Abstract 2

Solid-state phenomena like disorder, polymorphism but also the occurrence of high-*Z*′ crystal structures can be linked via energy differences in ‘archetype crystal structures’, which will permit better prediction of their occurrence.

## 1. Introduction

There is interest in the chemical and pharmaceutical industry (Deglmann *et al.*, 2015 ▸; Lam *et al.*, 2020 ▸) in convenient and efficient *ab initio* and semi-empirical computations, and this includes calculation of accurate solid-state properties from optimized experimental crystal-structure input today. Computational complexity increases when experimental crystal structures exhibit disorder, large unit cells, high-*Z*′, solvate formation, impurities and partially occupied atoms on crystallographically special positions – but not twinning, which is a macroscopic phenomenon. Though highly complex crystal structures (*e.g.* Feng *et al.*, 2012 ▸) are infrequent, they are regularly encountered in industry due to the large number of samples probed. In addition, the molecular sizes of active pharmaceutical ingredients (APIs) are growing with time (Baell *et al.*, 2013 ▸; Bryant *et al.*, 2019 ▸; Doak *et al.*, 2016 ▸); beyond-rule-of-five compounds (DeGoey *et al.*, 2018 ▸) are becoming more frequent. The average molecular mass for registered new drug molecules is increasing, and the larger conformational space of such molecules provides more opportunities for disorder. Industry processes therefore need to be robust enough to deal with crystal structures that are computationally challenging. Our motivation here is to conceptualize underlying factors of structural complexity via ‘archetype structures’ (Dittrich, Sever & Lübben, 2020 ▸ *b*) to gain experience with new methods for solid-state computation contributed earlier (Dittrich, Chan *et al.*, 2020 ▸ *a*), and to study their application to experimental input crystal structures that are challenging to compute.

### 1.1. Archetypes, high-*Z*′, disordered, polymorphic structures and solid solutions/cocrystals

An ‘archetype structure’ is a constituent that, when considering translational symmetry in the solid state, contributes to an average structure, which is what we observe experimentally by diffraction. In practical terms, it can be extracted from, for example, a disordered structure using one of several disordered parts (modelled by split positions) together with the non-disordered atoms (Dittrich, 2021 ▸). The concept of ‘archetype structure’ (Dittrich, Server *et al.*, 2020 ▸ *b*) can help to link disorder to polymorphism and solid solutions: when an archetype structure (as extracted from a disordered structure) can be obtained experimentally in pure form, we would consider it a polymorph. For polymorphic structures, the constituting molecule is the same. For solid solutions (Lusi, 2018 ▸) this is not the case, so here an archetype structure consists of one pure component in a crystal packing of a solid solution. Solid solutions are well defined as being maintained with changing ratios of components. In cases where a solid solution is only stable with a limited miscibility range, it might be better called a cocrystal.

**Figure 1:**
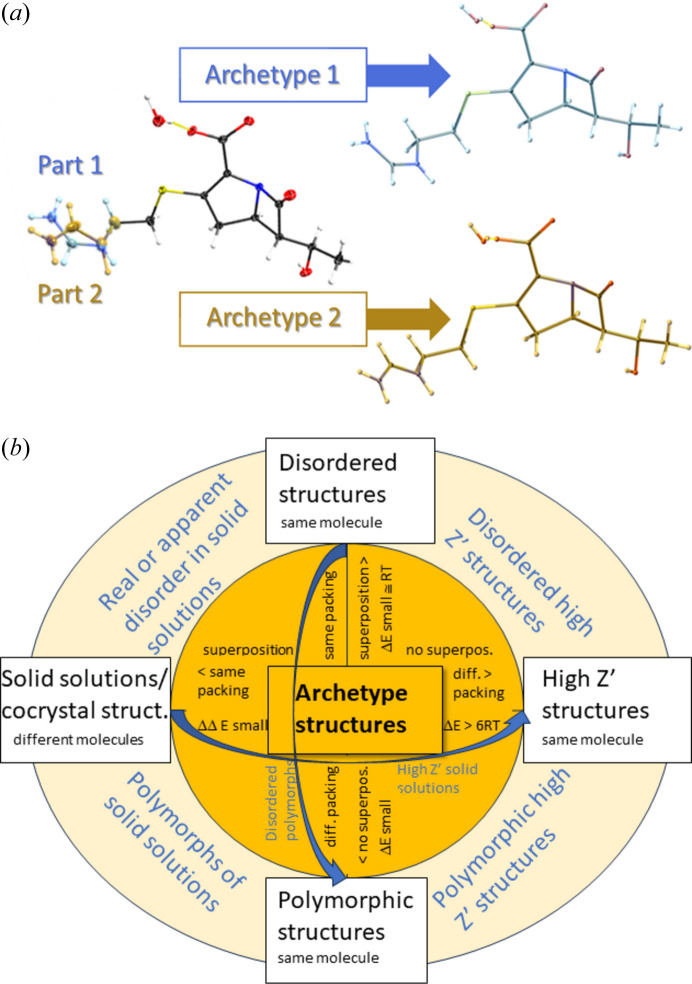
(*a*) Illustrating archetype structures extracted from the disordered structure of imipenem monohydrate. (*b*) Overview of solid-state phenomena linked by archetypes. On the vertical axis, disordered structures are a superposition of archetypes of similar energy, usually within small Δ*E* ≃ *R·T*, whereas polymorphs are distinct crystal structures, also with a small Δ*E* between them. We note that a similar Δ*E* rationale will also be underlying phase transitions.

An overview of how archetype structures link different solid-state phenomena via energy differences is given in Fig. 1 ▸. For simple two-component disorder with all atoms on general positions, two archetypes can be extracted. Concerning their small relative energy differences, archetype structures provide a rationale for why disorder occurs (Dittrich, 2021 ▸) together with other relevant criteria (Dittrich *et al.*, 2018 ▸; Müller *et al.*, 2006 ▸). After QM optimization of contributing archetypes, a disordered average structure can easily be reconstructed for experimental least-squares refinement, with archetype-specific restraints allowing us to obtain chemically consistent, accurate and precise results. One can easily see that considering entire molecules, including both ordered and disordered atoms together in separate archetypes, is essential for computation.

**Figure 2:**
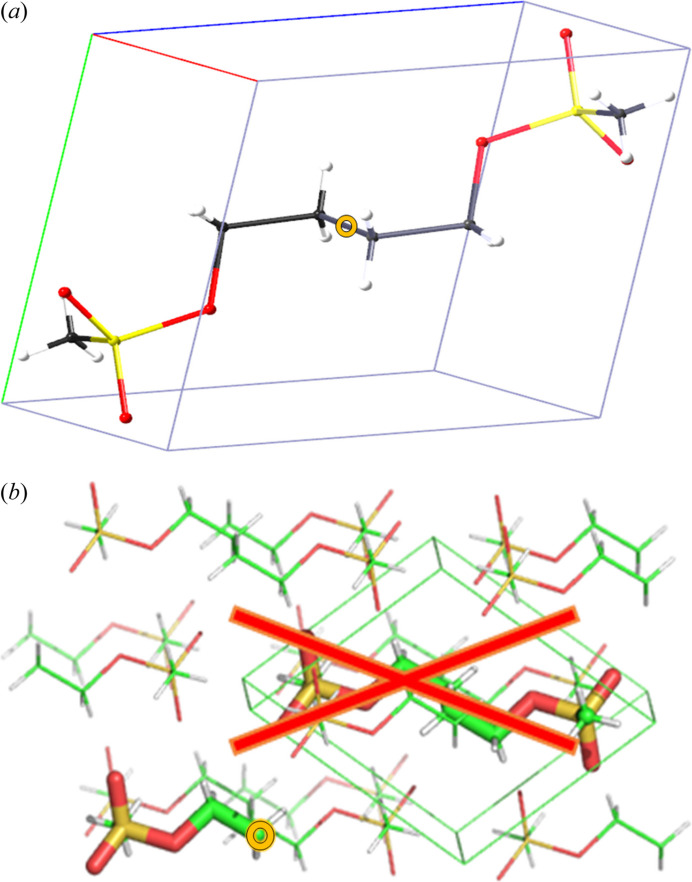
(*a*) Structure of KADKIJ, illustrating that computation requires an entire molecule and SG change from *P* 1 to *P*1 to generate a valid cluster of complete molecules, unlike in (*b*), a cluster consisting of half molecules. The centre of inversion is highlighted.

The initial need to consider archetypes thus stems from computation. When computing a crystal structure of a larger organic molecule, using *PIXEL* (Gavezzotti, 2002 ▸, 2003 ▸; Reeves *et al.*, 2020 ▸) and other related non-periodic cluster approaches (Dittrich, Chan *et al.*, 2020 ▸ *a*; Thomas *et al.*, 2018 ▸), space group (SG) and molecular symmetry need to be disentangled [*e.g.* when a symmetric (solvent) molecule resides on a crystallographic special position]. Computation then requires lowering of SG symmetry affecting the smallest unit of a crystal, a molecule or ion pair. This can be illustrated with the structure of busulfan [Fig. 2 ▸; CSD refcode KADKIJ (Taylor & Wood, 2019 ▸; Groom *et al.*, 2016 ▸)], where SG symmetry needed to be changed from *P* 1 to *P*1 and where molecular inversion and SG inversion centres coincide.

Here we are interested in extending applicability of archetypes to other complex phenomena encountered in crystal structure elucidation,^1^ refinement and computation. First, we look at structures with atoms of partial molecules residing on crystallographic special positions, where the rest of the asymmetric unit (ASU) atoms/ions/molecules do not appear to be disordered. Then we include high-*Z*′ structures, where archetype structures can contribute to their description and understanding.

### 1.2. Archetypes and structures with atoms on special positions

Like disordered structures with atoms on general positions, crystal structures containing (fully or partially occupied) solvent on special positions can be described by an overlay of archetype structures where molecular symmetry and SG symmetry coincide within the resolution of the experiment. Archetype structures are a simple means to disentangle a structure into contributions to the average structure for computation, but they can also be considered as representing macroscopic domains. Domains in turn are thus conveniently computed by clusters of alike ASU repeat units, corresponding to correlated disorder, but more work is required to show how often assuming correlation is valid. Sometimes a lower-symmetry subgroup of an experimentally observed average structure of higher symmetry [an aristotype (Müller, 2013 ▸)] can be observed for the constituting archetype structures. Like disordered structures with atoms on general positions, these systems are a manifestation of averaging through diffraction. When solvent has partial occupancy, it is not uncommon to observe additional disorder over special positions. In such cases, the archetype ASU energies are similar but there can be a mismatch between molecular symmetry and SG symmetry. Computationally, MIC optimization (Dittrich, Chan *et al.*, 2020 ▸ *a*) and subsequent ONIOM (Svensson *et al.*, 1996 ▸) computation comparing high-layer energies is most suitable here since the experimental unit-cell parameters are known and maintained. A lower-symmetry subgroup of an experimentally found average structure of a higher-symmetry aristotype would then apply for the underlying archetype structures. We provide three selected illustrative examples from the drug subset (Bryant *et al.*, 2019 ▸) of the CSD in the Results[Sec sec3].

### 1.3. Extending application: archetype structures extracted from high-*Z*′ structures

The concept of ‘archetype structure’ can be applied to explain high-*Z*′ structures. Such structures can show stunning complexity and have gained continuous interest throughout the last decades (*e.g.* Brock, 2016 ▸; Steed & Steed, 2015 ▸; Lehmler *et al.*, 2002 ▸; Rekis *et al.*, 2021 ▸; Pratt Brock & Duncan, 1994 ▸; Desiraju, 2007 ▸; Chandran & Nangia, 2006 ▸; Babu & Nangia, 2007 ▸; Roy *et al.*, 2006 ▸; Nichol & Clegg, 2006 ▸, 2007 ▸; Clegg, 2019 ▸). To simplify, we focus on three examples of *Z*′ = 2 structures and apply a procedure related to lowering the symmetry of archetype structures as outlined in Section 1.2, but in reverse: we generate an archetype with a smaller unit cell [related to Brock’s pseudocell (Duncan *et al.*, 2002 ▸)] with just one molecule in the ASU rather than two, optimize the hypothetical archetype structure and compare its molecule-pair interactions (Dittrich *et al.*, 2023 ▸). To generate the hypothetical one-conformer crystal structure from a structure with two distinct conformations, we start from both molecules in the ASU and focus on the lower-energy result. Halving the *a*, *b* or *c* lattice constant provides a packing of molecules with the same conformation. In analogy to the adjustment energy (Cruz-Cabeza & Bernstein, 2014 ▸), where a local gas-phase energy minimum close to the solid-state conformation is compared with a solid-state conformation, we compare an energy gain for an ordered *Z*′ = 2 structure with respect to a *Z*′ = 1 energy. A molecular conformational adjustment energy does not need to be subtracted since the lattice energy can be compared directly. This requires optimizing the real *Z*′ = 2 as well as the single-conformer *Z*′ = 1 archetype structures including lattice constants, since these can now change considerably, so periodic computations (Kühne *et al.*, 2020 ▸) are best suited to optimize these archetype structures. We propose the packing adjustment energy to be the major contributor to the formation of high-*Z*′ structures, since *Z*′ adjustment energy gain underlies or is related to several earlier explanations or observations: (1) packing problems, *e.g.* due to directional versus non-directional interactions (Pratt Brock & Duncan, 1994 ▸); (2) interaction frustration, deduced from the considerable number of high-*Z*′ structures obtained from sublimation crystallization (Clegg, 2019 ▸; Nichol & Clegg, 2006 ▸, 2007 ▸); (3) high-*Z*′ structures may be described as ordered modulated structures (Chandran & Nangia, 2006 ▸; Hao *et al.*, 2005 ▸); (4) the presence of many equi-energetic conformations co-existing in a crystal (Roy *et al.*, 2006 ▸); (5) pseudosymmetry (Görbitz & Torgersen, 1999 ▸; Lehmler *et al.*, 2002 ▸); (6) incomplete crystallization, ‘fossil relics of a crystal on the way to the thermodynamic minimum’, Gibbs–Helmholtz enthalpic balancing of entropic contributions (Desiraju, 2007 ▸).

Rather than qualitative descriptions, the recipe of calculating the *Z*′ adjustment energy gains can quantitatively show how unfavourable and destabilizing it can be to maintain only one rather than two or several conformations, explaining why increasing structural complexity can stabilize crystal packing.

### 1.4. Archetype structures and twinning

‘Twins are regular aggregates consisting of crystals of the same species joined together in some definite mutual orientation’ (Giacovazzo *et al.*, 1992 ▸), the orientation being described by a twin law. A twin law applies to macroscopic domains and is not part of SG symmetry [*e.g.* for merohedral twins, the twin law is a symmetry operator of the crystal system, but not of the point group of the crystal (Herbst-Irmer & Sheldrick, 1998 ▸)]. In contrast to a twin law, molecular symmetry can be included in SG symmetry. We can thus rank symmetry in a diffraction pattern into a hierarchy of molecular, SG and inter-domain contributions. Only the first two are relevant for computation of an ‘archetype crystal structure’. Since it is the differing orientation of an entire domain that causes twinning, and since twin domains are represented by the same archetype structure, twinning does not complicate *ab initio* (periodic or cluster) computation. Twinning is a macroscopic effect, special-position and high-*Z*′ structures require analysis on the molecular scale.

## 2. Methods

### 2.1. Data retrieval of structures studied

**Figure 3:**
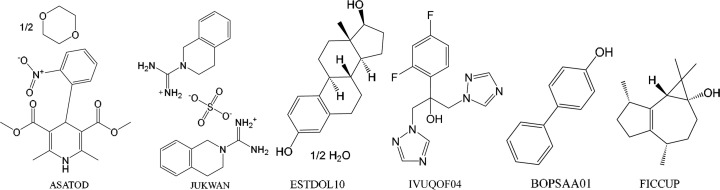
Lewis structures of the ASU content of the crystal structures studied, with CSD refcodes given below.

Structures investigated were downloaded from the Cambridge Structural Database (CSD) (Taylor & Wood, 2019 ▸) as Protein Data Bank PDB files for import into *BAERLAUCH* file format. ASU molecules in these structures and their CSD refcodes (Fig. 3 ▸) are nifedipine 1,4-dioxane clathrate (ASATOD), debrisoquinium sulfate, (JUKWAN), 17β-oestradiol (ESTDOL10), fluconazole (IVUQOF04), 4-hydroxy­biphenyl (BOPSAA01) and 1*Z*,2*R*,4*R*,7*S*,11*S*-3,3,7,11-tetra­methyl­tri­cyclo­[6.3.0.0^2,4^]undec-1(8)-en-4-ol (FICCUP).

### 2.2. Computational tools and procedures

**Figure 4:**
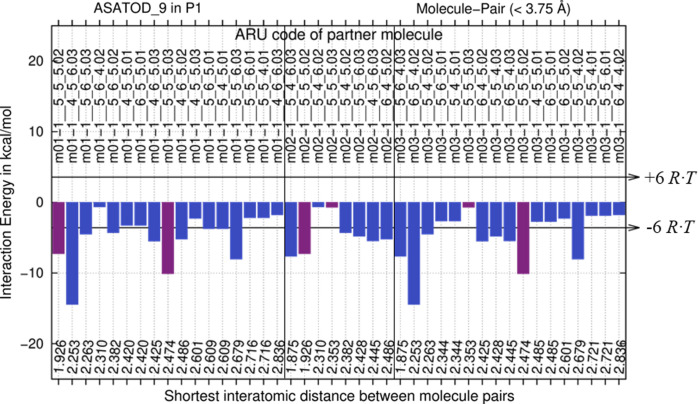
E(MPIE) analysis of nifedipine, the symmetry of which has been lowered to enable computation.

Retrieved crystal structures were initially optimized using the molecule-in-cluster (MIC) (Dittrich, Chan *et al.*, 2020 ▸ *a*) approach, generating clusters of molecules around a central ASU. When unit-cell parameters required optimization, full-periodic computation followed. The semiempirical quantum mechanical method GFN2-xTB as implemented in the *XTB* program (Bannwarth *et al.*, 2019 ▸) or GFN1-xTB in *CP2K* (Kühne *et al.*, 2020 ▸) was relied upon. More accurate single-point energies for the ASU or the chemical system specified were obtained by two layer MO:MO (molecular orbital) ONIOM (Svensson *et al.*, 1996 ▸) methods using dispersion-corrected density functional theory (DFT-D) and the APFD functional (Austin *et al.*, 2012 ▸) with the 6-31G(d,p) basis set for the high layer, and 3-21G for the low layer with the *Gaussian* program (Frisch *et al.*, 2016 ▸). Although the double-zeta Pople basis is incomplete (Moran *et al.*, 2006 ▸), it was chosen for its computational efficiency in our single-point computations, for limiting basis-set superposition, and having application to larger pharmaceutical molecules in mind. For the JUKWAN structure, charge, spin and multiplicity for high and low layers needed manual specification, respectively, all other input was automatically generated by the pre-processor *BAERLAUCH* (Dittrich *et al.*, 2012 ▸) from ASU content. A distance threshold of 3.75 Å between ASU atoms and symmetry-generated atoms (completing those molecules whose atoms are within the atom–atom threshold distance) gives suitable clusters (Dittrich *et al.*, 2012 ▸), and was chosen throughout for MIC computations, whereas one entire unit cell content provided input for full-periodic computations with *CP2K*. Charged/ionic species like those present in JUKWAN often require larger distance thresholds, or use of implicit solvation models (*e.g.* Ehlert *et al.*, 2021 ▸) for MIC optimization, but were not required here. Hypothetical archetype structures used for studying high-*Z*′ structures required optimization of unit-cell parameters and these were optimized by *CP2K* (version 2023.2), maintaining SG symmetry. Here the DFT-3 dispersion correction with BJ damping was used (Grimme *et al.*, 2011 ▸). The Gaussian plus plane wave computation used defaults for grid levels and 300 a.u. for the first and second, and for further levels 100, 33.3, 11.1 and 3.7 a.u. as density cut-off. Selected optimized structures (see below) were subsequently evaluated by molecule-pair interaction energies E(MPIE) as introduced recently (Dittrich *et al.*, 2023 ▸). Here, a ‘3.75 Å cluster’ is divided into pairs of molecules always containing a central ASU molecule with the symmetry code 1__5_5_5.01 (Spek, 2003 ▸) and a symmetry-generated partner molecule, whose symmetry is provided in the E(MPIE) plot (*e.g.* Fig. 4 ▸). In that analysis, the pairwise interaction energy is calculated by subtracting molecular single-point energies from the molecule-pair GFN2-xTB single-point energy, maintaining the crystal geometry. E(MPIE) energies are then ordered according to the shortest intermolecular atom–atom distance, keeping track of partner-molecule symmetry operation and translation. A good guide to interpret so-obtained energies is a ± 6 *R·T* threshold: when a molecule-pair interaction exceeds 6 *R·T* (Thomas *et al.*, 2018 ▸), it is unlikely that the crystal structure is stable. This threshold can also help in identifying structure determinants (Gavezzotti & Filippini, 1995 ▸). Only when a stabilizing energy is below −6 *R·T* would we consider it important in the formation of a crystal packing. A 6 *R·T* threshold thus helps to assess the likelihood of a structure being experimentally accessible, and whether optimization gives plausible results. Like the PIXEL approach (Gavezzotti, 2003 ▸, 2002 ▸), E(MPIE) analysis shares the advantage that interactions between entire molecules (Carlucci & Gavezzotti, 2005 ▸) are considered as in the literature (Moggach *et al.*, 2015 ▸; Reeves *et al.*, 2020 ▸; Maloney *et al.*, 2014 ▸), not only those of functional groups (Etter, 1990 ▸). Book-keeping symmetry and subtracting energies of pairwise energy computation with *XTB* was performed with *BAERLAUCH* (Dittrich *et al.*, 2012 ▸).

## 3. Results and discussion

Disordered crystal structures and archetypes have already been discussed in detail (Dittrich, 2021 ▸). Recapitulating that the energy difference of analogous ASU content of archetypes forming a disordered average structure is within very tight bounds, usually within *R·T*; we are next interested if this finding also applies to systems where SG symmetry and molecular symmetry are intertwined.

### 3.1. Archetypes structures with atoms on special positions

#### 3.1.1. 1,4-Dioxane clathrate (Nifedipine) in *P* 1

The first system studied is nifedipine with half a dioxane solvent on a special position (CSD refcode ASATOD). Cluster computation of the structure required an SG change from *P* 1 to *P*1. In *P*1, three molecules in the ASU were generated from 1.5 molecules in *P* 1, all of which were MIC-optimized using the entire ASU content. *PLATON* (Spek, 2003 ▸, 2009 ▸) and the ADDSYM routine find that nifedipine molecules are superimposable before and after optimization within the assumed thresholds. Hence, changing the SG back to *P* 1 would be possible after QM analysis. Fig. 4 ▸ shows the E(MPIE) evaluation of the three independent molecules in the (artificial) ASU used for computation.

**Figure 5:**
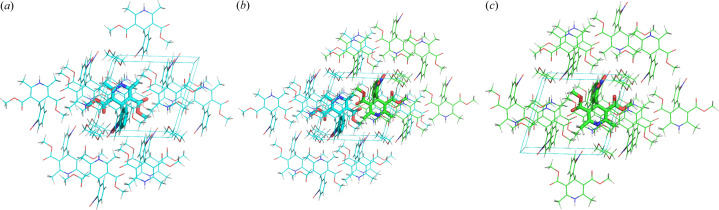
Symmetry-generated local cluster input for nifedipine (*a*) molecule three (blue) and its counterpart (*c*) molecule one (green) with their local cluster environments and ASU cluster, and (*b*) a combination of the two.

The summary bar-plots from E(MPIE) analysis need further explanation. There are three molecules *m*01, *m*02 and *m*03 and their clusters are summarized at once in Fig. 4 ▸. The left part of the plot shows molecule 1 (symmetry codes starting with *m*01), the middle molecule is the dioxane solvent (*m*02) and the right molecule is the symmetry-generated third molecule that can be superimposed with *m*01. The bars show molecule-pair interaction energies and permit identification of strong intermolecular interactions, as identifiable by the symmetry operation generating the second molecule in the pair (*e.g.* 1__5_5_6.03). Bars in the bar-plot are ordered by the shortest interatomic distances between atoms in molecule pairs. It can be seen from these shortest distances that interactions of *m*03 are not identical to *m*01 after optimization in *P*1. While the local cluster environments (Fig. 5 ▸) conform exactly to lower *P*1 SG symmetry, and share the same number of molecules, their optimized coordinates deviate from higher symmetry and do not ‘perfectly’ superpose to *P* 1. Like in quasicrystals, small deviations from perfect symmetry are possible – and still lead to diffraction. Despite such small differences, local clusters show very similar intermolecular interaction energies. Computations that are more sophisticated may reduce deviations, but the point is that experimental symmetry does not need to be fulfilled perfectly. Symmetry informs us about energetic similarity within an energy window. Horizontal lines indicate the 6 *R·T* threshold to distinguish strong from weak intermolecular molecule-pair interactions. Interactions between molecules within the ASU are coloured magenta, interactions to molecules outside are blue. As stated, interactions in E(MPIE) plots are ordered by shortest distance. This illustrates that the stabilization observed from wavefunctions interacting is not distance-dependent, showing that a focus on proximity of functional groups (*e.g.* hydrogen bonding) is the wrong paradigm. It can lead to missing the relevance of other intermolecular interactions like π-stacking, where distances do not easily permit estimating strength from interatomic distances.

Concerning computational robustness, basis set superposition error (BSSE) is not as relevant in GFN2-xTB (Bannwarth *et al.*, 2019 ▸) as in more sophisticated DFT-D computations. We have verified (not shown) that the trends in the most significant pairwise energy gains are very similar when performing slower, but more accurate DFT-D computations.

We further analysed the ASU molecules using ONIOM computations. In this method (Svensson *et al.*, 1996 ▸) the cluster is partitioned into a high layer (treated with a higher-level computational method) and a low layer (less good but faster method of theory), the high layer being the ASU molecule, the low layer the cluster environment (Fig. 5 ▸). From the high-layer energies we can see that the two independent molecules generated from changing SG indeed do not differ by much. The APFD/6-31G(d,p):APFD/3-21G^2^ computation shows an energy difference of just 2.4 kJ mol^−1^,^3^ very close to *R·T*. The cluster input of the two local crystal environments is shown in Fig. 5 ▸. Since energies in the blue and the green cluster around molecules 1 and 3 are very similar, and within the energy available at *R·T* during crystallization,^4^ the average structure can be formed as a superposition of two archetype crystal structures. Consequently, a higher *P* 1 symmetry is observed from the *P*1 archetype structures. The presence of symmetry indicates their energetic similarity within the energy available during crystallization. We expect that optimization and analysis at higher levels of theory provide even smaller energy differences. Optimization itself might even induce noise but is required as the case of oestradiol below will show.

#### 3.1.2. Debrisoquinium sulfate in *C*2/*c*

For the crystal structure of CSD refcode JUKWAN, debrisoquinium sulfate or bis­[3,4-di­hydro-2(1*H*)-iso­quinoline­carboxamidinium] sulfate, there is initially one main positively charged molecule and half a sulfate dianion on a special position in the CCDC deposition. Computation of the structure requires an SG change from *C*2/*c* to *P*2_1_/*n*. Like for nifedipine, the structure is not disordered, and we can consider it being composed of two archetypes that overlay within the resolution of the experiment to give an average structure of higher symmetry. Lowering symmetry as for nifedipine generates a structure with three ions in the ASU that was again MIC optimized. The E(MPIE) plot (not shown) is dominated by ionic interactions. Just considering symmetry we would expect the energy of the two now independent molecules to be the same. This is confirmed by the ONIOM computation, where with 3.4 kJ mol^−1^ *R·T* with *T* = 298 K is slightly exceeded. This is probably because we use a distance cut-off in the cluster; for the predominantly ionic interactions in this structure, a gas-phase calculation of a cluster is probably not best suited to capture these interactions. Increasing the cluster size was not attempted, since the 3.75 A shortest atom–atom threshold including whole molecules already led to a reasonable cluster size. Trying an additional PCM continuous solvent environment of this cluster led to a slightly larger energy difference. Still, we consider this energy difference being in the right ballpark concerning *R·T*. There are numerous other examples in the CSD of structures like nifedipine or debrisoquinium sulfate with ions or solvent on special positions, and we think that providing computational details for two of them, while not statistically significant, is sufficient to explain them. A statistical analysis using the DFT-D level of theory, while being out of scope, would be desirable to further support energetic findings.

#### 3.1.3. β-Oestradiol in *P*2_1_2_1_2

**Figure 6:**
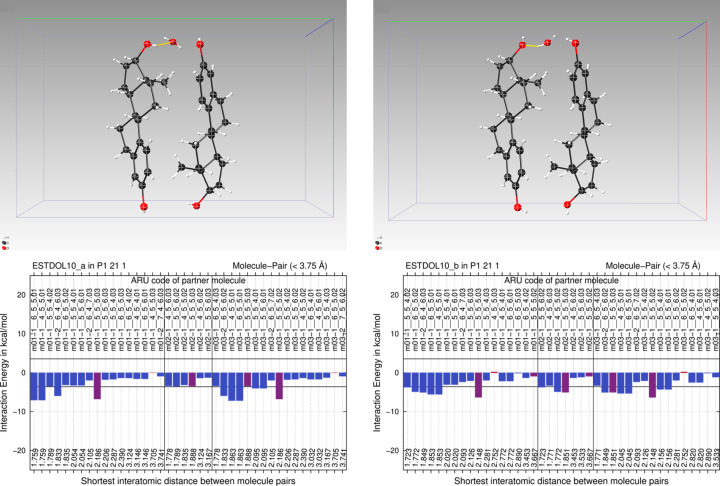
E(MPIE) analysis of the two archetypes present at once in the average structure of oestradiol. Adding up the individual contributions shows that the left hydrogen bond pattern is, with 55 kcal mol^−1^, less stabilizing than the other (right) 68 kcal mol^−1^.

Studying the structure of oestradiol hemihydrate (CSD refcode ESTDOL10) provides further insight beyond debrisoquinium sulfate or nifedipine. Computation of the crystal structure again requires an SG change, here from *P*2_1_2_1_2 to *P*2_1_, giving three ASU molecules each in two archetype crystal structures. Notably, their optimization leads to different hydrogen-bonding patterns (Fig. 6 ▸), and all OH and water protons must therefore be disordered in the experimental average structure, which we confirmed in a refinement with deposited structure factors. ESTDOL refcode depositions contain either only one incomplete or a mixed set of hydrogen atoms.

In contrast to nifedipine, *PLATON* ADDSYM thus does not suggest an SG change back to *P*2_1_2_1_2 for the *P*2_1_ archetypes due to non-superposable oxygen and hydrogen atom positions. This leads to the question of whether strictly speaking the assigned SG is correct. Here there is no easy answer. From a practical perspective, structure refinement fails (also including tight structure-specific restraints) in overlaying both lower-symmetry SG *P*2_1_ archetype structures, since only the signal of disordered hydrogen atoms breaks the *P*2_1_2_1_2 symmetry. This signal is very small compared with the ‘ordered’ (or better superimposable) part of the structure. Since non-hydrogen atoms superpose nicely, *P*2_1_2_1_2 seems correct. However, the *P*2_1_2_1_2 average structure leads to the wrong charge density of this structure and is chemically incorrect. This structure is thus unsuited for charge density analysis (Koritsánszky & Coppens, 2001 ▸) or quantum crystallographic X-ray wavefunction refinements (Davidson *et al.*, 2022 ▸; Jayatilaka, 1998 ▸), since the experimental electron density it provides in *P*2_1_2_1_2 is an overlay of two archetypes with a different hydrogen-bonding pattern. Distinguishing the hydrogen-bonding patterns by how energetically favourable they are is possible. Interestingly, these do not equally contribute to an average structure. The caption of Fig. 6 ▸ shows a summation (adding individual energy gains) of molecule pairs, indicating that they are rather different at the GFN2-xTB level of theory considering only molecule pairs. Although the two archetype structures lead to similar molecular conformations, one hydrogen-bonded network is energetically more stabilizing.

**Figure 7:**
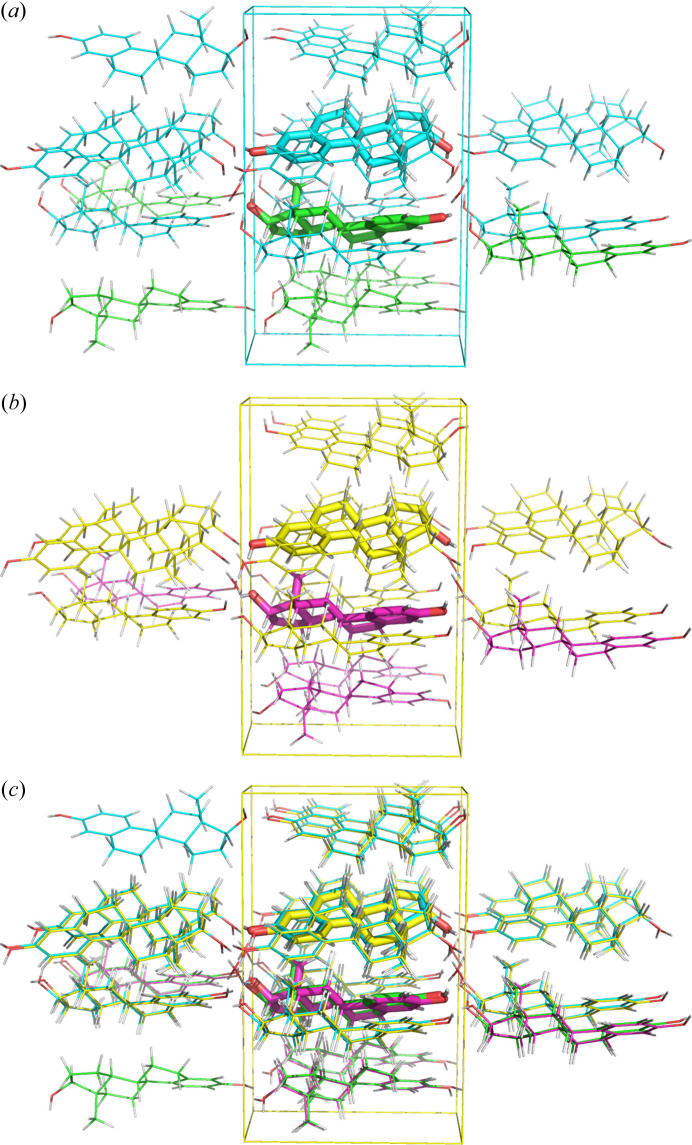
(*a*) and (*b*) Local clusters contributing to the (*c*) average structure of oestradiol.

More accurate DFT-D analysis confirms that archetype crystal structure ONIOM high-layer energies differ: the two pairs of main molecules plus water from different clusters that form the average structure (Fig. 7 ▸, bottom) can be ordered by high-layer energy and are ranked 7.1, 3.4 and 3.4 kJ mol^−1^ (−850.1976, −850.2003, −850.2018 Hartrees) with respect to the lowest energy (−850.2031 Hartrees). This leads us to speculate that local domains of hydrogen-bonded patterns might also be different in solution, and that they are not easily switched in liquid or solid due to the barrier exceeding *R·T*. Exceeding *R·T* as observed in the average structure is due to dynamically swapping hydrogen atoms which increase the entropy of the system. An additive entropy contribution to *R·T* (Thomas *et al.*, 2018 ▸) should thus be considered in systems like oestradiol. 6 *R·T* as an upper limit should in general not be exceeded.

### 3.2. Solid solutions

For solid solutions it is harder to evaluate computational results since molecules/ion pairs in archetype crystal structures differ (Fig. 1 ▸). Energy differences between them can thus not be interpreted easily. However, one could calculate an energy difference between two states, for example gas phase and solid state in a ΔΔ*G* approach for drawing conclusions. Our hypothesis (Fig. 1 ▸) is that the energy gain from packing is very similar for both archetype structures in direction and magnitude also when molecule A is in a molecule B environment, and B is in an A environment. This will be the subject of a future investigation.

### 3.3. High-*Z*′ structures

After considering how archetype structures provide clarity in analysing special-position crystal structures and in relating them to polymorphism (composition of alike molecules) and solid solutions (composed of different molecules) in Fig. 1 ▸, next examples of *Z*′ = 2 structures were studied. The question we try to answer is why these molecules crystallize in a high-*Z*′ arrangement with two different conformations, and not in a single-conformation *Z*′ = 1 packing. To permit statistical analyses of archetype-structure energy differences, many more structures would need to be studied. This is beyond the scope of the current paper, where we propose an analysis framework. For analysis of the following three crystal structures, the archetype analysis strategy is applied in reverse, providing a workflow how to analyse high-*Z*′ structures in general. With *Z*′ increasing, so does the number of possible archetype structures. Energy differences between them will then guide us which ones are relevant. The following simple examples of *Z*′ = 2 structures are orthorhombic, with the same kind of symmetry in each direction, facilitating the splitting of the unit cell into two fragments for separate optimization.

#### 3.3.1. Fluconazole in *Pbca*

**Figure 8:**
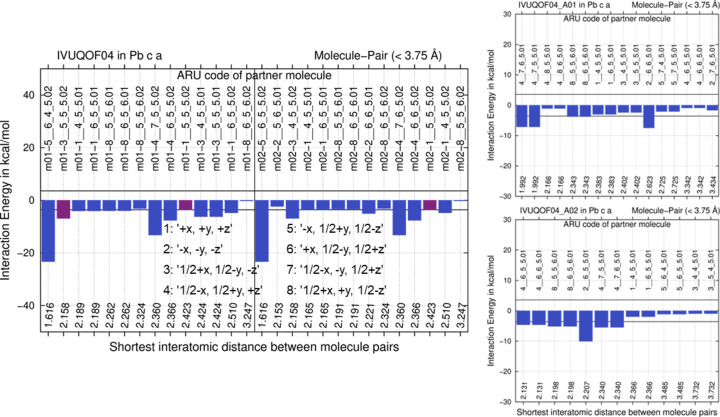
E(MPIE) GFN2-xTB evaluations of the *CP2K* GFN1-xTB optimized structures. Left: starting from the fluconazole experimental *Z*′ = 2 crystal structure IVUQOF04; right: comparison with the two optimized hypothetical archetype structures generated from it. Symmetry operations of SG *Pbca* used in ARU codes given in both E(MPIE) plots are provided in the left insert. Intermolecular interactions involving the molecules in the ASU are given in magenta, others in blue.

For fluconazole CSD crystal structure IVUQOF04 in the SG *Pbca*, we maintain SG symmetry and generate two hypothetical *Z*′ = 1 archetype structures by halving the *Z*′ = 2 unit cell in the *b* axis direction. Since conformations differ, each of two molecules in the ASU of an experimental *Z*′ = 2 structure leads to an archetype.^5^ The experimental crystal structure and their hypothetical archetype crystal structures were then optimized by a full-periodic GFN1-xTB approach with the program *CP2K*, providing impressive speedup to pioneering earlier DFT-D work (Van De Streek & Neumann, 2010 ▸) – at lower accuracy. Optimized coordinates are subsequently analysed in terms of GFN1-xTB lattice-energy differences, and via GFN2-XTB E(MPIE) plots. We note that there is conceptually no difference between a trial structure generated in a crystal structure prediction run (Schmidt & Englert, 1996 ▸; Price, 2004 ▸; Neumann & Van de Streek, 2018 ▸) and an archetype in this context. However, we would not consider all CSP trial structures archetype structures; a relationship to an experimental crystal structure which was proven to exist is required in our opinion. In this context, E(MPIE) and the 6 *R·T* criterion might prove useful for filtering un-realistic trial structures, and to understand whether they can exist; lattice-energy differences for fluconazole structure IVUQOF04 (Fig. 8 ▸) and the archetype structures generated from it are +78.8 and +323.1 kJ mol^−1^ per molecule. The two hypothetical *Z*′ = 1 archetype structures have fewer stabilizing interactions exceeding a 6 *R·T* threshold, whereas the E(MPIE) plot for the optimized experimental structure shows a dominating, ‘structure determining’ (Gavezzotti & Filippini, 1995 ▸) strong intermolecular interaction (Fig. 8 ▸, left). The directionality of the interactions and the energetic driving force for the *Z*′ = 2 structure can thus conveniently be identified and visualized from E(MPIE) analysis and quantified by lattice-energy differences.

#### 3.3.2. 4-Hy­droxy­biphenyl in *P*2_1_2_1_2_1_

**Figure 9:**
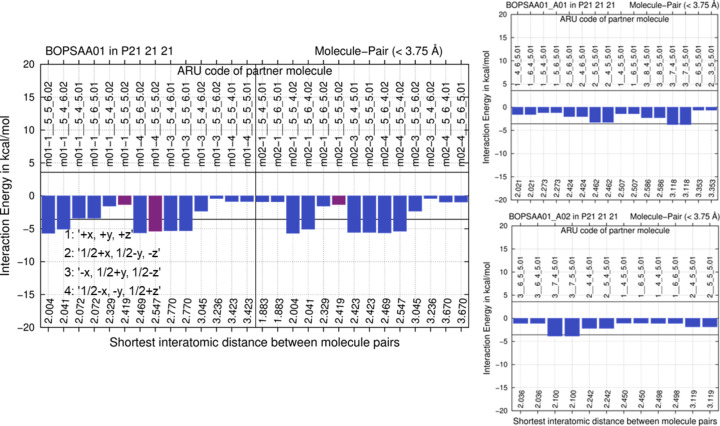
E(MPIE) GFN2-xTB evaluation of the GFN2-xTB optimized experimental *Z*′ = 2 crystal structure in comparison with the lower energy of two DFT-D optimized hypothetical archetype structures generated from the experimental 4-hy­droxy­biphenyl structure BOPSAA01. Symmetry operations of SG *P*2_1_2_1_2_1_ used in ARU codes underlying both E(MPIE) plots are provided in the left insert.

The same computational and archetype-structure generation and analysis strategy was applied to the 4-hy­droxy­biphenyl crystal structure with CSD refcode BOPSAA01, (Brock & Haller, 1983 ▸), but the level of theory needed to be increased. To generate two hypothetical archetype structures the unit cell was halved in the *c* direction in this case. Like before, results show that having only one rather than two conformations in the ASU leads to an energy penalty, since hydrogen bonding of the hy­droxy group is impossible in a *Z*′ = 1 structure (Fig. 9 ▸).

The experimental (average) crystal structure gives a planar molecule. Torsion energy differences for bi­phenyl between planar and non-planar conformations are within *R·T* (Sancho-García & Cornil, 2005 ▸). Semi-empirical GFN2-xTB MIC optimization of the experimental structure leads to small deviations from planarity. Neither is hy­droxy­biphenyl planarity computationally reproduced with *CP2K* at the semi-empirical GFN1-xTB level of theory. Hence, we optimized unit-cell parameters and structure of the experimental starting structure, as well as the derived archetype structures, also at the Gaussian plus plane wave PBE/DZVP level of theory^6^ (Krack, 2005 ▸; VandeVondele *et al.*, 2005 ▸), with the above-mentioned GD3BJ dispersion correction that was also applied in GFN1-xTB with *CP2K*. The better DFT-D level of theory indeed results in overall planar coordinates; however, for the experimental structure BOPSAA01 optimization provided a denser, lower-symmetry structure with SG *P*2_1_ and *Z*′ = 4, with *PLATON* identifying pseudosymmetry. Since this optimized structure is not directly comparable anymore, in Fig. 9 ▸ (left) the non-planar GFN2-xTB MIC optimization result is reported, where experimental *Z*′ = 2 and SG *P*2_1_2_1_2_1_ are maintained. However, relative energy differences are reported at the DFT-D level of theory, *i.e.* between the archetype structures and the *P*2_1_ result. They remain broadly comparable to FICCUP below and IVUQOF04 above; they are +13.4 and +26.5 kJ mol^−1^ per molecule, significantly above 6 *R·T*. Concerning E(MPIE) analysis with GFN2-XTB, *Z*′ = 1 archetype structures have no significant stabilizing interactions (Fig. 9 ▸, right). Even in the GFN2-xTB E(MPIE) analysis of BOPSAA01 grown from sublimation (Fig. 9 ▸ left), there are not that many favourable interactions when compared with the other experimental structures (Fig. 3 ▸). The lack of stabilizing interactions in the *Z*′ = 1 archetype structures, *e.g.* those connected with the ARU code 3__5_4_6 (symmetry operation 3 given in the left part of Fig. 9 ▸ with a translation of −1 in the *b* and +1 in the *c* direction), shows that neither hypothetical structure should crystallize under ambient conditions. This example also illustrates the driving force of higher *Z*′ crystal structure formation.

#### 3.3.3. 1*Z*,2*R*,4*R*,7*S*,11*S*-3,3,7,11-Tetramethyl­tri­cyclo­[6.3.0.0^2,4^]undec-1(8)-en-4-ol in *P*2_1_2_1_2_1_

**Figure 10:**
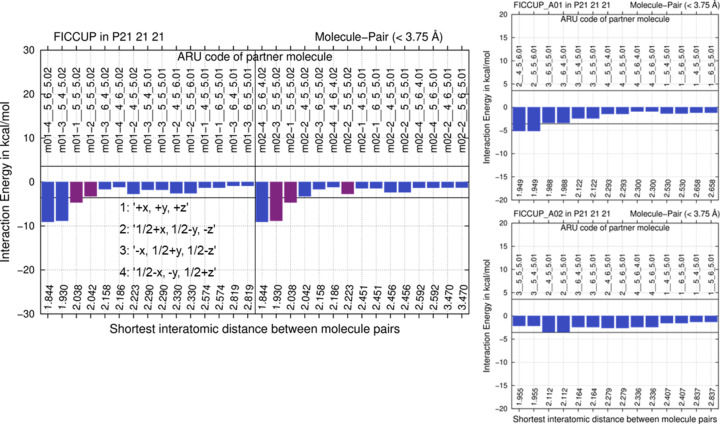
E(MPIE) GFN2-xTB evaluation of the GFN1-xTB optimized experimental *Z*′ = 2 crystal structure in comparison with the two hypothetical archetype structures generated from the experimental 1Z,2R,4R,7S,11S-3,3,7,11-tetra-methyl­tri­cyclo­(6.3.0.0^2,4^)undec-1(8)-en-4-ol structure FICCUP. Symmetry operations of SG *P*2_1_2_1_2_1_ used in ARU codes given in both E(MPIE) plots are provided in the left insert.

For CSD refcode crystal structure FICCUP analysis results using the semi-empirical computational strategy likewise show that a *Z*′ = 1 structure is energetically less stabilizing than an experimental *Z*′ = 2 arrangement. Again, there are few stabilizing interactions in the optimized lower-energy archetype structures (right side of Fig. 10 ▸) above the −6 *R·T* threshold and this is an indication to not make them plausible experimental crystal structures. There are at least *n* possibilities when generating archetype structures from *Z*′ = *n* structures (increasing combinatorically with *n*); we again focused on the more stabilizing lower-energy ones.

The GFN1-xTB lattice-energy differences between the experimental and the lower-energy hypothetical *Z*′ = 1 archetype structures are +14.4 and +13.4 kJ mol^−1^. As in the two cases before, the energy gain from a *Z*′ = 2 structure, or the penalty of a single conformer *Z*′ = 1 crystal structure is higher than the 6 *R·T* criterion proposed. While due to pronounced energy differences fluconazole was obviously not crystallizing in the two *Z*′ = 1 archetype crystal structures directly related to the experimental structure IVUQOF04, energy differences for BOPSAA01 and FICCUP and the archetypes extracted get closer to 6 *R·T*. When energy differences become even smaller, the region where the full variety of solid-state phenomena is encountered gets closer (Fig. 1 ▸).

## 4. Conclusions and outlook

An ‘archetype crystal structure’ was introduced in the study of imipenem monohydrate, where it became apparent that disorder, solid solutions and polymorphism are closely related (Dittrich, Server *et al.*, 2020 ▸ *b*). Disorder was found to be attributed to very similar energies of archetype structures, a finding confirmed for crystal structures with atoms on special positions in this work, namely nifedipine and debrisoquinium sulfate, but not estradiol, where an entropy contribution adding to enthalpy is the suspected cause. In this work, we have made the approximation not to take entropy into account. Typical Gibbs free energy differences due to molecular vibrations were quantified to be up to 7 kJ mol^−1^ (Nyman & Day, 2015 ▸) between polymorphs, and we will try to include such contributions in future work. A promising approach would be computing (molecular) entropies as additional correction factors (Grimme, 2019 ▸) on the same GFN2-xTB level of theory as used above. Considering special-position structures as being composed of overlaying archetypes provides a recipe for computational treatment. Archetype crystal structures also aid our understanding and the analysis of the formation of high-*Z*′ structures (Fig. 1 ▸), where comparison of hypothetical *Z*′ = 1 and experimental *Z*′ = 2 archetypes illustrates why higher-*Z*′ structures lead to more stabilizing crystal packings. Rather than enigmatic crystal structures, or ‘crystals on the way’, high-*Z*′ structures maximize stabilizing intermolecular interactions by conformational or orientational change. Overall, archetypes are key to better understand the ‘average structure’ observed by experiment. Classification of polymorphs, disordered structures, solid solutions and high-*Z*′ structures according to ONIOM or lattice-energy differences shows that crystallographic symmetry and energy are mutually dependent (related to Noether theorem): if crystallographic symmetry is observed experimentally, symmetry-related molecules have approximately the same energy, within ranges available at crystallizing conditions; high-*Z*′ structures show reduced local symmetry to avoid an energetic penalty.

## Footnotes

1 It is conceivable that isostructurality (Bombicz, 2024 ▸, 2017 ▸) might likewise be categorizable through energy differences.

2 For two-layer ONIOM with force-field embedding, bigger basis sets can be used, but can lead to BSSE (not quantified). For our APFD/6-31G(d,p):APFD/3-21G MO/MO computation the CPU time is significantly larger than for FF treatment of the low layer. The use of Pople bases in comparing relative energy differences is therefore merited.

3 It is good advice to be sceptical about the energy accuracy of semi-empirical DFT-B and also two-layer DFT-D ONIOM computations; in the text two significant figures are provided.

4 Changing the crystallization temperature can shift the 6 *R·T* cut-off significantly. Given that some compounds crystallize in the same form at 20, 0 and −80°C, whereas others change form at lower or higher *T*, 6 *R·T* provides guidance of the importance or limitations of changing *T* (or *p*) to obtain different forms. *e.g.* we haven’t seen de-stabilizing E(MPIE) exceeding + 6 *R·T* in structures containing neutral molecules so far.

5 When only the hydrogen bonding differs but the molecular conformation is the same, one still has two possibilities to generate archetypes. When molecular conformations differ considerably, so that a pair forms the entity to crystallize together as in refcode CEKBAV, for example, the reverse archetype analysis strategy obviously fails.

6 There are obviously many other functionals to choose from. PBE was chosen since it was used extensively and successfully in crystal structure prediction blind tests.
